# Benefits of Prenatal Taurine Supplementation in Preventing the Onset of Acute Damage in the Mdx Mouse

**DOI:** 10.1371/currents.md.9a3e357a0154d01050b591601cbd4fdb

**Published:** 2017-09-22

**Authors:** Robert G. Barker, Deanna Horvath, Chris van der Poel, Robyn M. Murphy

**Affiliations:** Department of Biochemistry and Genetics, La Trobe Institute for Molecular Science, Melbourne, Victoria, Australia; Department of Physiology, Anatomy, and Microbiology, La Trobe University, Melbourne, Victoria, Australia; Department of Physiology, Anatomy, and Microbiology, La Trobe University, Melbourne, Victoria, Australia; Department of Biochemistry and Genetics, La Trobe Institute for Molecular Science, Melbourne, Victoria, Australia

## Abstract

**Introduction:**

Duchenne Muscular Dystrophy (DMD) is a debilitating muscle wasting disorder with no cure. Safer supplements and therapies are needed to improve the severity of symptoms, as severe side effects are associated with the only effective treatment, corticosteroids. The amino acid taurine has shown promise in ameliorating dystrophic symptoms in mdx mice, an animal model of DMD, however little work is in 21-28 (d)ay animals, the period of natural peak damage.

**Methods:**

This study compares the effect of prenatal taurine supplementation on tibialis anterior (TA) in situ contractile function, histopathological characteristics and the abundance of Ca^2+^-handling as well as pathologically relevant proteins in non-exercised mdx mice at 28 and 70 d.

**Results:**

Supplementation elevated TA taurine content by 25% (p<0.05), ameliorated in situ specific force by 60% (p<0.05) and improved histological characteristics in 28 d mdx mice; however no benefit was seen in 70 d mice, where background pathology was initially stable. Age specific effects in SERCA1, calsequestrin 1 (CSQ1), CSQ2, utrophin and myogenin protein abundances were seen between both 28 and 70 d mdx and mdx taurine-supplemented mice.

**Discussion:**

Considering these findings and that taurine is a relatively cost effective, readily accessible and side effect free dietary supplement, we propose further investigation into taurine supplementation during pregnancy in a protective capacity, reminiscent of folate in the prevention of spinal bifida.

## Introduction

Duchenne muscular dystrophy (DMD) is a fatal, X-linked neuromuscular disorder affecting approximately 1:3500-6000 live male births^1^^,^^2^. DMD is caused by a mutation in the dystrophin gene which results in the absence of the cytoskeletal protein dystrophin. The dystrophic pathology is most notably characterised by progressively debilitating muscle weakness, as cumulative bouts of muscle degeneration exhaust regenerative capabilities and healthy myofibers are replaced by non-contractile, fat and fibrotic tissue. Ultimately this loss of dystrophin results in the loss of ambulation, respiratory complications, and lethal cardiac events by approximately 30 years of age^3^^,^^4^. There is currently no cure for DMD, and while genome editing using CRISPR/Cas9 technologies are showing great promise for future treatment^5^ there remains a persistent need for safer alternatives to current treatments utilising steroids^1^, which present the patient with a host of adverse side effects.

Dystrophin is part of a network of proteins known as the dystrophin-glycoprotein complex (DGC) that anchor the contractile machinery of a myofiber to the extracellular matrix. Whilst fundamentally this provides the muscle with stability during contraction there is increasing evidence that dystrophin, as a part of the DGC, also plays a role in cell signalling. Therefore while the precise mechanisms by which dystrophin deficiency leads to the muscle weakness seen in DMD remains unclear; it is clearly multi-faceted in nature^6^. The most common animal model of DMD is the dystrophin deficient mdx mouse, which has a premature stop codon in exon 23 of the dystrophin gene^7^. In this model, as well as other animal models, altered Ca^2+^ homeostasis; increased reactive oxygen species; inflammation and membrane destabilisation have all been implicated in the pathology of the disease^6^.

Importantly, the mdx mouse does not experience the progressive muscle wasting that leads to death in DMD patients^8^^,^^9^. Rather mdx mice experience an acute onset of hindlimb muscle necrosis from 21-28 (d)ays, at which time they most closely represent the severity of muscle pathology seen in DMD, before stabilising into low grade but persistent muscle damage into adulthood (>10 weeks). The up-regulation of utrophin, the structural homolog of dystrophin, as well as the cessation of growth and exercise avoidance behaviours all contribute to the milder phenotype observed in adult mdx mice^9^. Critically, however, DMD patients exhibit a more severe dystropathology, and whilst in response to initial bouts of damage and/or necrosis DMD patients experience myogenesis and regeneration, in contrast to the mdx mouse, this persists for years as opposed to weeks. The relatively stable pathology found in adult mdx mice can be exacerbated with damaging exercise protocols, and although this can be a source of inconsistency between studies provides a useful platform for the investigation of therapeutic interventions. However, the adult model does not allow the investigation of interventions that would act in a preventative or protective capacity to the acute phase of muscle degeneration, such as that observed naturally in the 21-28 d mdx mouse. Preventative strategies aimed at reducing the onset of degeneration, as opposed to those attempting repair when damage is overwhelming may show promise in alleviating dystrophic symptoms if the disease is diagnosed early.

Taurine, a sulphur containing amino acid, is found ubiquitously throughout the excitable tissues of the body where it is associated with healthy muscle function, differentiation and growth (For review see^10^^,^^11^). It is available commercially as a dietary supplement and is widely used as an ergogenic aid^12^. Of note, its use has no documented obvious side effects when supplemented in humans^12^. In healthy animals and mdx mice, taurine supplementation has been found to improve Ca^2+^ handling, membrane stabilisation, oxidative stress and inflammation both at rest and following damaging exercise protocols^13^^,^^14^^,^^15^. Recent investigations reported similar muscle taurine content in juvenile mdx mice at most ages (e.g. 18, 22, 36 and 42 days) compared to WT mice, although reduced in 28 d mdx mice^13^^,^^16^^,^^17^. These findings support that taurine levels vary with age in the growing mdx mouse. It appears that during the onset of mdx pathology taurine supplementation is efficacious at preventing the myofiber necrosis and inflammation in juvenile mdx mice if elevated early^13^. Of relevance, normal (non-dystrophic) mice deficient in the taurine transporter (TauT), as well as those with dramatically reduced intramuscular taurine content, experience dystrophic symptoms including compromised muscle strength, susceptibility to fatigue, and developmental abnormalities^18^^,^^19^. Taurine supplementation has been previously studied in vivo and in vitro in mdx mice, typically following chronic exercise protocols, finding both supplementation and direct application to muscle preparations effective at improving muscle health and force development^16^^,^^20^.

This study aims to investigate the efficacy of taurine at ameliorating dystrophic symptoms in the mdx mouse at two distinct pathological stages (28 and 70 d). Taurine was supplemented prenatally and throughout the life of animals to ensure the maximum effect of the amino acid. This study addresses the importance of age as a key consideration for pathological relevance when screening for therapeutic supplements, as well as when investigating biochemical and physiological properties of muscle from mdx mice. Our results show that at 28 d, during the onset of acute muscle damage, taurine was effective at increasing muscle strength and improving visual muscle health but shows no benefit to 70 d mdx mice where the background pathology is initially stable.

## Materials & Methods

**2.1 Animals and supplementation**

All procedures in this study were approved by the La Trobe University Animal Ethics Committee (AEC 12-31, 13-48). Only male mice were used for experiments. A total of 35 mdx and 24 wild-type (WT, C57/BL10ScSn) were used. Experimental animals were bred at the La Trobe Animal Research and Teaching Facility using breeding pairs obtained from the Animal Resource Centre (Western Australia, Australia). The offspring of WT and mdx mice had access to standard rodent chow, water ad libitum and were utilised for experimentation at either 28 ±1 or 70 ±1 days of age. Mdx taurine (mdx tau) breeders and subsequent offspring were supplemented with continuous access to taurine (2.5% wt/vol) enriched drinking water, with breeders beginning supplementation at least two weeks prior to mating. This dosage of supplementation has been demonstrated previously to elevate skeletal muscle taurine content by up to 40% when given directly to rats^21^. The number of animals used in the various experiments sometimes differ due to sedation issues that occurred during in situ experimentation. This required the animal to be immediately culled.

**2.2 Muscle Dissection**

Mice were anesthetized with an initial intraperitoneal injection of 10ul.g^-1^ Nembutal (Sodium Pentobarbitone) and kept unresponsive for the duration of the experiment with supplementary doses (10% initial volume). The mouse was weighed, placed on a 37^o^C heated pad and the right leg was skinned to the waist taking care not to damage the fascia. Throughout the experiment, warmed physiological saline (0.9%) was applied to exposed muscle tissue. The distal tendon of the tibialis anterior (TA) muscle was isolated and secured with both a top and bottom knot as close to the myotendinous junction as possible using 5.0 surgical thread (Ethicon, Johnson & Johnson, NSW, Australia). The distal tendon was then severed and the TA dissected free from the surrounding tissue keeping nerve and blood flow intact at the tibia below the tibial plateau. The sciatic nerve was exposed by gently parting the midline of the biceps femoris muscle above the knee joint. The mouse was secured on the heated platform (37^o^C) of an in situ contractile apparatus (809B in situ Mouse Apparatus, Aurora Scientific, Ontario, Canada) with a pin behind the patella tendon and a foot clamp. The distal end of the TA was tied firmly to a lever arm attached to an isometric force transducer. The sciatic nerve was stimulated by two field stimulating platinum electrodes coupled to an amplifier.

**2.3 Contractile Protocol**

The TA was contracted via square wave (0.2 ms) pulses at 10V from the stimulator (701C stimulator, Aurora Scientific, Ontario, Canada). Forces were converted to a digital signal and recorded by Dynamic muscle analysis 611A (DMA) (Aurora Scientific, Ontario, Canada). Optimum muscle length (L_o_) was first determined by eliciting twitch contractions at incrementally adjusting muscle length with a micromanipulator until a repeatable maximum peak twitch force (Pt) was obtained. Muscle length at L_o_ was measured with precision digital callipers from the beginning of the distal tendon to the insertion of the TA at the base of the knee. Subsequently the TA was stimulated at 100 Hz tetanic contraction, followed by a 2 min rest interval and then twitch contraction. Comparable twitch forces pre and post 100 Hz stimulation indicated that the knots were both secure and unlikely to slip during the remaining protocol. If a decrease in twitch force was observed the muscle was incrementally tensioned and stimulated between 2 min rest intervals until P_t_ was re-established. To establish the force frequency relationship the TA was stimulated for 500 ms at 10, 20, 30, 40, 50, 80, 100, 150 and 200 Hz with a 2 min rest interval in-between. Maximum isometric tetanic (P_o_) force was determined from the largest force produced during the force-frequency stimulation protocol. Maximum tetanic specific force (sP_o_) was determined as force per cross sectional area (CSA) as described by Lynch (DMD_M.2.2.005). Briefly, optimum fiber length (Lf) was first determined by multiplying L_o_ by the predetermined TA length to fiber length ratio of 0.6^22^ and utilising the formula sP_o_ = P_o_ x (muscle mass/Lf x 1.06).

Following the force frequency protocol, the TA was allowed a 2 min recovery prior to commencement of the fatigue protocol. Fatigue was induced by stimulating the TA for 500 ms at 60 Hz every second for 180 s. At the completion of the fatigue protocol, recovery was assessed by stimulating the muscle for 500 ms at 60 Hz following 1, 2 and 3 min recovery periods.

Immediately following the contraction protocol, the mouse was removed from the apparatus and the contractile TA dissected, blotted clean on filter paper (Whatman No.1) and weighed, before being embedded in O.C.T. compound (Tissue-tek) and snap frozen in liquid nitrogen cooled isopentane (2-methylbutane, Sigma Aldrich). The contralateral TA was also collected and snap frozen. All muscles were stored at -80°C until analysis. The mouse was then killed by cardiac excision.

**2.4 Taurine content analysis**

The contralateral TA was freeze dried and powdered. Approximately two milligrams was powdered in a mortar and pestle and added to 20 % (wt: vol) sulfosalicyclic acid solution (Sigma Aldrich), vortexed and placed on ice. Samples were then centrifuged at 12,000 g 2^o^C for 2 min. Supernatant (125 μl) was collected, added to 900 μl of 0.4 M borate buffer and placed on ice before centrifuged at 12,000 g 2^o^C for a further 2 min. Supernatant (850 μl) was removed and stored at -80°C for HPLC analysis, which was carried out by ACS Laboratories (Kensington, Australia). Samples were analysed along with a series of standards (r^2^ = 0.99) and peak area was used to express taurine concentrations as μmol.g^-1^.

**2.5 Muscle Histology**

Transverse cryosections (8 μm) were cut from the midpoint of the TA and mounted on positively charged microscope slides (Lomb Scientific). Slides were stained with haematoxylin and eosin (H&E) and images were taken using a Motic BA310 microscope mounted with a Moticam 5 camera running Motic image plus 2.0 software (Motic, China). A semi-quantitative approach was taken on the contracted limb of four animals from each group, determining non-contractile area (i.e. area containing no contractile machinery including but not limited to necrotic myofibers, fragmented sarcoplasm, connective tissue and inflammatory cells) as a percentage of the total area of the section, total number of fibers per section and the number of fibers containing central nuclei. All analysis was performed blind by four parties and performed interactively, both manually and using Image J software.

**2.6 Western Blotting**

Frozen TA cryosections were cut from the midpoint (~30 x 10 µm 28 d animals, ~20 x 10 µm 70 d animals) and immediately placed into 1X SDS solution (3X SDS solution (0.125 M Tris-HCI, 10% glycerol, 4% SDS, 4 M urea, 10% mercaptoethanol and 0.001% bromophenol blue, pH 6.8) diluted 2:1 with 1× Tris.Cl (pH 6.8), 5mM EGTA). Samples incubated for at least 30 min at room temperature, vortexed at five minute intervals and stored at -80°C until analysed. Aliquots of each TA sample were pooled together and used to create a calibration curve that was run on every gel, allowing comparisons of whole muscle homogenates across gels^23^^,^^24^. Total protein from each sample was initially separated on 4-15% gradient Criterion TGX Stain Free gels (BioRad, Hercules, CA) and following UV activation using a Stain Free Imager (BioRad), the densities of the total lanes was obtained (Image lab software v 5.2, BioRad) and used to ensure equal loading for subsequent western blotting.

Quantitative western blotting was performed to determine the protein abundance of actin, myosin heavy chain 1 (MHC1), ryanodine receptor (RyR), sarco/endoplasmic reticulum Ca^2+^ ATPase pump 1 (SERCA1), dihydropyridine receptor (DHPR), Calsequestrin 1 (CSQ1) and 2 (CSQ2), dystrophin, utrophin, myogenin and taurine transporter (TauT). The western blotting protocol was similar to that described previously^25^^,^^26^^,^^27^. Briefly, an equal amount of protein from TA samples as well as a four to five point calibration curve was separated on 4-15% gradient Criterion TGX Stain Free gels (BioRad, Hercules, CA). All samples from WT, mdx and mdx tau mice were run in at least duplicate for each protein (except MHC1) and averaged across gels. Prior to transfer gels were imaged with a Stain Free Imager (BioRad) for total protein which was quantified for each sample (Image lab software v 5.2, BioRad). Following this, using a wet transfer protocol, protein was transferred onto a nitrocellulose membrane at 100V for 30 min. Following transfer the gel was imaged again and the membrane incubated in Pierce Miser solution (Pierce, Rockford, IL) for ~10 min and then blocked in 5% skim milk powder in 1% Tris-buffered, saline-Tween (TBST) for ~2 h at room temperature. Following blocking membranes were incubated in primary antibodies overnight at 4°C and 2 h at room temperature. All antibodies were diluted in 1% bovine serum albumin (BSA) in phosphate buffered saline (PBS) with 0.025% Tween (PBST).

Antibody details and dilutions are as follows: rabbit polyclonal monoclonal anti-Actin (Batch A 2066, SIGMA, 1:300); mouse monoclonal anti-MHC1 (A4.840, Developmental Studies Hybridoma Bank (DSHB), IgM, 1:200), mouse monoclonal anti-RyR1 (34C, DSHB, 1:300), mouse monoclonal anti-SERCA1 (CaF2-5D2, DSHB, 1:1,000), mouse monoclonal anti-DHPR (IIID5E1, DSHB, 1:400), mouse monoclonal anti-CSQ1 (ab2824, Abcam, 1:2000), rabbit polyclonal anti-CSQ2, (ab3516, Abcam, 1:1,000), rabbit polyclonal anti-Taurine Transporter (TauT, Professor David Pow, RMIT University, 1:10,000), mouse monoclonal anti-Utrophin (Mancho3 clone 8A4, DSHB, 1:200), mouse monoclonal anti-Dystrophin (MANDYS1 clone 3B7, DSHB, 1:500) and mouse monoclonal anti-Myogenin (F5D, DSHB, 1:250). The gel of protein extracts from mdx muscle was probed for dystrophin to confirm the absence of this protein.

After washing, membranes were incubated with a secondary antibody (goat anti-mouse IgG or IgM, goat anti-rabbit IgG, HRP conjugated, 1:60,000) and rinsed in TBST. Bands were visualized using West Femto chemiluminescent substrate (ThermoScientific, IL, USA) with images taken and densitometry performed using Image Lab software (BioRad). The positions of molecular mass markers were captured under white light before chemiluminescent imaging, without moving the membrane. Total protein and specific protein densities were each expressed relative to their respective calibration curves and subsequently each protein was normalised to the total protein content (25).

**2.7 Statistics**

All data are presented as mean + standard deviation (SD) unless stated otherwise. Comparisons between WT and mdx, mdx and mdx tau groups were performed using a 1-way ANOVA of variance with individual stimulation frequencies in the force-frequency analysis, and individual time points in the fatigue and recovery analyses, with Sidak’s post-hoc analyses. All statistical analysis was performed using GraphPad Prism v 6. Significance was set at p<0.05.

## Results

**3.1 Effect of taurine supplementation on body mass, muscle mass, TauT protein content and taurine content**

Taurine supplementation (in utero and in the drinking water) had no effect on the body mass of 28 d or 70 d WT, mdx and mdx tau mice (Table 1). The TA muscle mass was similar in 70 d mdx and mdx tau mice, and was 23% greater in the mdx mice than the WT mice (p<0.05, Table 1). At 28 d no difference in TA muscle mass was observed between WT, mdx and mdx tau groups (Table 1).

**Figure:**
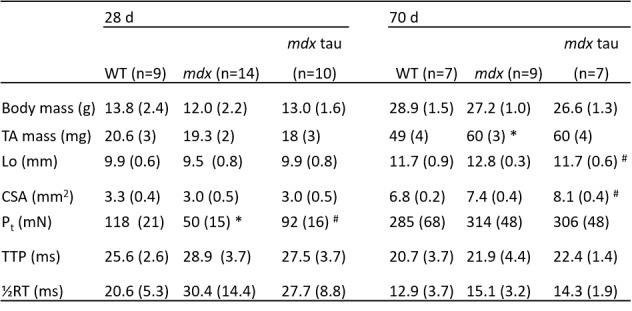
**Table 1:** Body mass, tibialis anterior (TA) mass, morphological, twitch and tetanic contractile properties of TA muscles from 28 and 70 d WT, mdx and mdx taurine supplemented (mdx tau) mice. Optimal length (Lo), cross sectional area (CSA), peak twitch force (Pt), time to peak tension (TTP) and half relaxation time (½RT). n = number of mice. Values are mean (SD); One way ANOVA with Sidak’s post-hoc analyses between age groups. Symbols for significant differences (p < 0.05) are: *significantly different from WT group, # significantly different than mdx group.

To determine the content of taurine transporter protein (TauT), over a range of loading amounts, the density of a known amount of total protein and TauT (indicated in Fig. 1A and 1B) was plotted as a calibration curve and individual samples were expressed relative to this calibration curve. As can be seen, the density of the TauT was low, however the use of the calibration curve meant that such amounts could be determined. There was no difference in the TauT abundance in the TA muscle across age or phenotype (Fig. 1C).

Muscle taurine content was measured in the TA muscle of the contralateral, non-stimulated limb of mice from each group. In the 28 d group mdx tau supplemented mice had 25% greater muscle taurine content (37 ±5 μmol.g^-1^) than non-supplemented mdx mice (28 ±2 μmol.g^-1^), and mdx mice taurine content was similar to WT mice (30 ±4 μmol.g^-1^, Fig 1D). Conversely at 70 d taurine supplementation proved ineffective, with mdx tau mice (39 ±7 μmol.g^-1^) having 22% less intramuscular taurine than the non-supplemented 70 d mdx group (47 ±6 μmol.g^-1^, p<0.05), and the mdx mice had similarly higher taurine levels than the 70 d WT mice (37 ±3 μmol.g^-1^). A significant age-specific increase in taurine content was observed with non-supplemented mdx mice increasing by 67% from 28 to 70 d (p<0.05). WT and mdx tau mice had no increase in taurine content with age ( Fig. 1D).

**Taurine transporter protein and taurine content in tibialis anterior muscles from 28 d and 70 d wild-type, mdx and mdx taurine supplemented mice.**
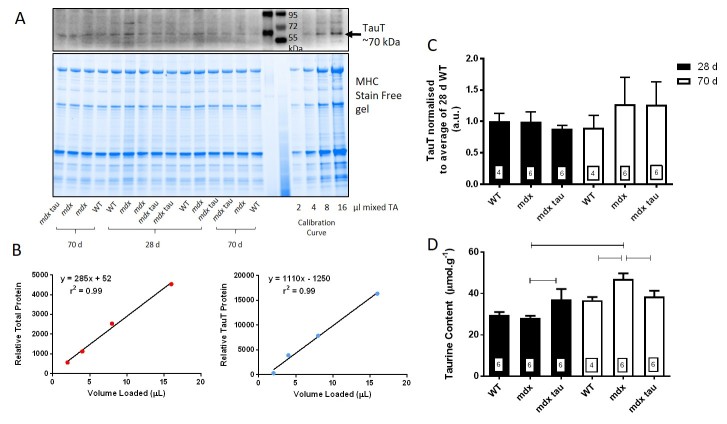
**A.** Western blot of taurine transporter protein (TauT, top) and Stain Free gel showing total protein (bottom) from tibialis anterior (TA) 28 and 70 d wild-type (WT), mdx and mdx taurine supplemented (mdx tau) mice. The four point calibration curve is indicated. Two different molecular weight markers are shown between the samples and the calibration curve, with the protein sizes indicated. **B.** Densities of total protein (left) and TauT protein (right) plotted against the calibration curve volumes loaded. The linear regression, y = mx + c is shown, along with correlation coefficient (r^2^). **C.** The TauT protein abundance is shown for 28 d (black bars) and 70 d (white bars) WT, mdx and mdx tau mice. **D.** TA taurine content represented as in C. Lines above specific bars indicate significant difference between groups (p<0.05). One way ANOVA with Sidak’s post-hoc analyses. Data presented as means + SD with n indicated in respective bars.

**3.2 Effect of taurine supplementation on twitch and tetanic contractile properties**

Figure 2A and Bshow the mean responses for in situ maximum and relative contractile force, respectively, for all groups. Table 1 shows the mean values for in situ twitch and tetanic TA muscle characteristics. While both maximum and specific forces in 28 d mdx mice were significantly weaker than the WT group, taurine supplementation ameliorated this weakness by approximately 60% when relative to TA muscle mass compared to the non-supplemented mdx mice (Fig. 2A, B). Whilst maximum force production was not significantly different between 70 d groups (Fig. 2A), when normalised to TA muscle mass, mdx mice produced a similar specific force to mdx tau mice, but ~27% less force than the WT mice (Fig. 2B). At 70 d taurine proved ineffective at increasing specific force production despite increasing muscle CSA by 9% (Fig. 2A, Table 1). Compared with 28 d WT, mdx mice had ~50% reduced peak twitch forces (Pt), but this was attenuated in 28 d mdx tau mice (Table 1). No differences were observed in optimum length (Lo), cross section area (CSA), time to peak tension (TTP) and half-relaxation time (½ RT) between all 28 d groups (Table 1). In 70 d animals, no differences were observed in Pt, TTP or ½ RT, but compared with mdx mice, mdx tau mice had ~9% smaller Lo (Table 1).

**In situ force production in tibialis anterior muscles from 28 d and 70 d wild-type, mdx and mdx taurine supplemented mice.**
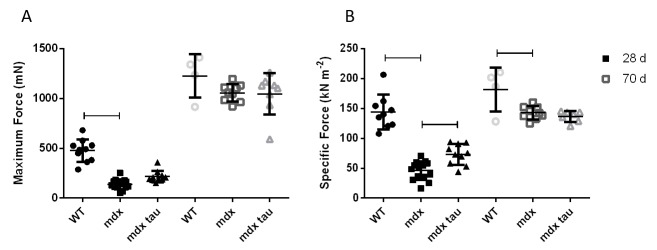
In situ maximum force (A) and specific force (B) productions in 28 d (solid symbols) and 70 d (hollow symbols) wild-type (WT), mdx and mdx taurine supplemented (mdx tau) mice. Lines above specific bars indicate significant differences between groups (p < 0.05). One way ANOVA with Sidak’s post-hoc analyses. Data presented as means + SD with n indicated.

**3.3 Force Frequency Relationship**

Despite taurine mice producing greater absolute forces (Fig. 2A), taurine had no effect on relative muscle force production at both low and high stimulation frequencies (p>0.05, Fig. 3). At 10 and 20 Hz, the TA muscle from mdx mice were more sensitive to the stimuli than WT animals, and similar to mdx tau mice, as indicated by producing a greater percentage of maximum force at these frequencies (Fig. 3). This trend persisted throughout the frequency range of 30 – 80Hz (Fig. 3). There was no significant difference in the force frequency curve relationship between 70 d groups (p>0.05, Fig. 3). Generally 28 d groups were more sensitive (i.e. produced a greater percentage of maximum force at a given frequency) across the lower physiological frequencies than 70 d groups. There was a significant age effect between mdx mice at 10, 20, 30, 40, 150 and 200Hz with the 28 d mdx mice more sensitive throughout (Fig. 3). Similarly 28 d mdx tau mice were more sensitive at 10, 20, 30, 40 and 50Hz when compared to the 70 d mdx tau group. 28 d WT mice were more linear throughout the frequency range than the 70 d WT.

**Force frequency relationship in 28 d and 70 d wild-type (WT), mdx and mdx taurine (mdx tau) mice.**
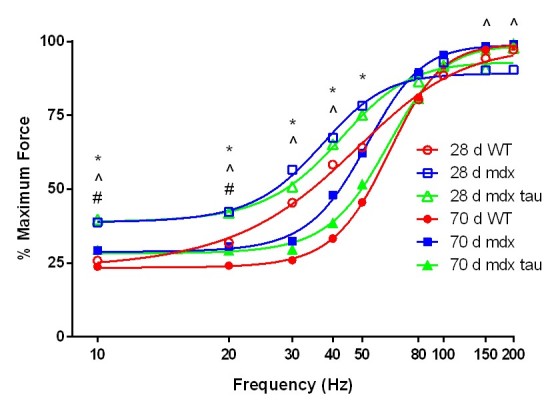
Data are presented as means, error bars have been removed for clarity (28 d WT n= 9, 28 d mdx n=14, 28 d mdx tau n=10, 70 d WT n=5, 70 d mdx n=9, 70 d mdx tau n=8). One way ANOVA with Sidak’s post-hoc analyses within relevant groups. Symbols for significant differences (p < 0.05) are: # 28 d WT vs. 28 d mdx, ^ 28 d mdx vs. 70 d mdx, * 28 d mdx tau vs. 70 d mdx tau.

**3.4 Fatigue Recovery**

In response to a low stimulation fatigue protocol, the 28 d WT mice experienced an approximately 40% reduction in force production after 180 s of in situ stimulation (Fig. 4-left). In response to the same protocol 28 d mdx and mdx tau mice fatigued significantly less than the WT, with an ~20% reduction in force production (Fig. 4-left). Following 180 s recovery, 28 d WT and mdx mice produced 120% of their pre-fatigue force and mdx tau mice produced 113%. These elevated forces persisted throughout all recovery intervals (Fig. 4-right). At 70 d there was no difference in the rate or degree of fatigue between groups, with WT, mdx and mdx tau groups producing 53%, 46% and 40% of their initial force, respectively (Fig. 4-left). There was no difference in force production at any recovery interval after the 180 s fatigue protocol in 70 d mice (Fig. 4-right).

**60 Hz fatigue and recovery in 28 d and 70 d wild-type (WT), mdx and mdx taurine (mdx tau) mice.**
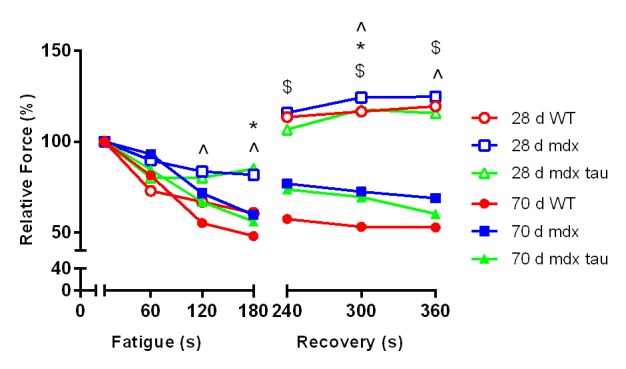
Data presented as means, error bars have been removed for clarity (28 d WT n= 9, 28 d mdx =14, 28 d mdx tau=10, 70 d WT=5, 70 d mdx=9, 70 d mdx tau n=8). One way ANOVA with Sidak’s post-hoc analyses within relevant groups. Symbols for significant differences (p<0.05) are: # 28 d WT vs. 28 d mdx, $ 28 d WT vs. 70 d WT, ^ 28 d mdx vs. 70 d mdx, * 28 d mdx tau vs. 70 d mdx tau.

**3.5 Histopathology**

To investigate the effect of age and taurine supplementation on muscle architecture the contracted TA was assessed in 28 and 70 d WT, mdx and mdx tau mice for markers of histopathology. Figure 5A shows representative transverse sections of each group. The area within each section that was comprised of non-contractile tissue (NCT) was quantified and expressed as a percentage (%) of total area (Fig. 5B). Total discernible muscle fibers were counted and the proportion of those that were centrally nucleated (CNF) was quantified (Fig. 5C). Approximately 22% of the TA cross sections from 28 d mdx mice was composed of necrotic and non-contractile tissue, with 55% of fibers being centrally nucleated and highly variable in diameter (Fig. 5). 28 d mdx tau mice had a marked reduction in histopathological features compared to the mdx mice, with ~50% reduction in NCT (Fig. 5B), a reduction in CNF (37%) and comparatively uniform muscle fiber diameter (Fig. 5C). Conversely, taurine had no effect at reducing the amount of NCT or CNF in 70 d mdx mice (Fig.s 5B, 5C), with both mdx groups visually exhibiting relatively uniform muscle fiber diameters reminiscent of the WT (Fig. 5A mdx tau 28 d and 70 d). Compared to the 28 d mdx mouse, there was both a natural 74% reduction in NCT and a marked visual improvement in fiber health in the 70 d mdx mouse. This age specific improvement was not evident in the WT or as pronounced in mdx tau groups.

**Histological characteristics of 28 d and 70 d wild-type, mdx and mdx taurine mice.**
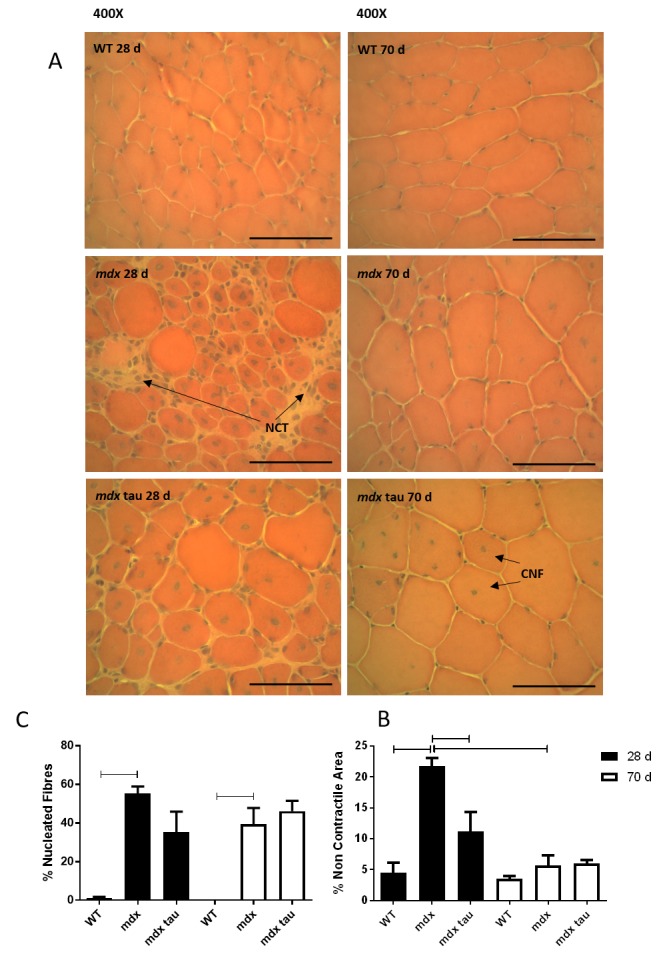
Representative haemotoxylin and eosin stained transverse sections of tibialis anterior muscles from 28 and 70 d WT, mdx and mdx tau mice shown at 400X magnification. Nuclei are stained dark, cytoplasm pink/orange. Fiber outlines are evident, non-contractile tissue (NCT) are indicated, and centrally nucleated fibers (CNF), scale bars = 100 µm. B. Quantification of the percentage area of NCT (B) and CNF (C) for 28 d (black bars) and 70 d (white bars) WT, mdx and mdx tau mice. Lines above specific bars indicate significant difference (p<0.05), One way ANOVA with Sidak’s post-hoc analyses. Data presented as means + SD, n = 4 all groups.

**3.6 Effect of taurine supplementation on abundance of Ca^2+^ handling proteins**

To investigate if the improvement in force with taurine supplementation could be attributed to altered Ca^2+^ handling, or the presence of an age specific effect, we measured the abundance of a number of relevant proteins, RyR1, DHPR, SERCA1, CSQ1, CSQ2, MHC1 and actin using quantitative western blotting (Table 2 and Fig.s 6 and 7). Taurine supplementation had no effect on the abundance of these proteins in either 28 or 70 d mdx mice, but there was an age specific affect in mdx and mdx tau mice, with an increase in the abundance of SERCA1 (51%) and a decrease in CSQ2 (65%) in 28 d mice and a 54% increase in CSQ1 in the mdx tau mice (Fig. 7).

**Representative blots for data shown in Table 2.**
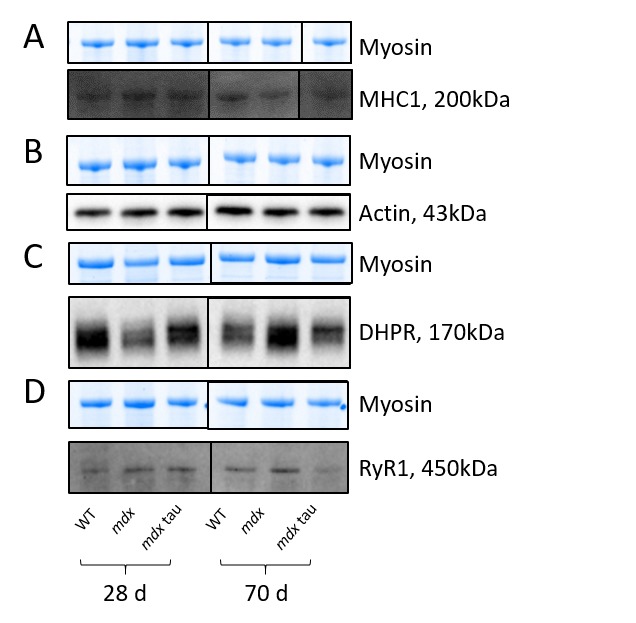
Shown for each panel is the myosin from the Stain Free gel, indicative of total protein (top) and the representative Western blot protein (bottom) for MHC1 (A, black line indicates non-contiguous lanes from the same gel), actin (B), DHPR (C) and RyR1 (D) in 28 d and 70 d WT, mdx and mdx tau mice.

**Figure:**
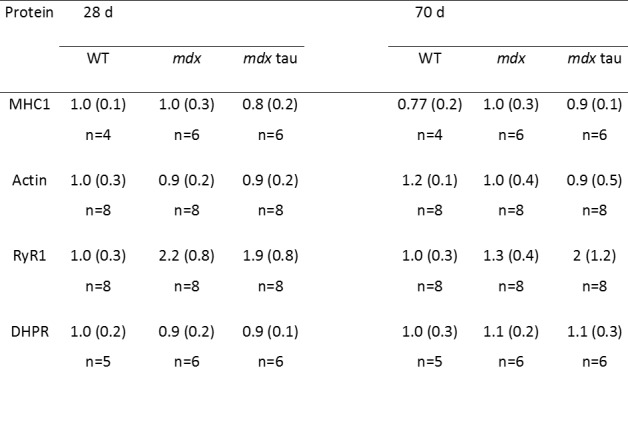
**Table 2:** Abundance of proteins important for excitation-contraction coupling in 28 d and 70 d wild-type (WT), mdx and mdx taurine (mdx tau) mice. Each protein is expressed relative to density of total protein determined from the Stain Free gel and then relative to the average of 28 d WT mice. One way ANOVA with Sidak’s post-hoc analyses between relevant groups. Significance at p<0.05, data presented as mean (SD) (no significant differences observed). Representative Blots shown in Figure 6.

**SERCA1 and calsequestrin in 28 and 70 d WT, mdx and mdx tau mice.**
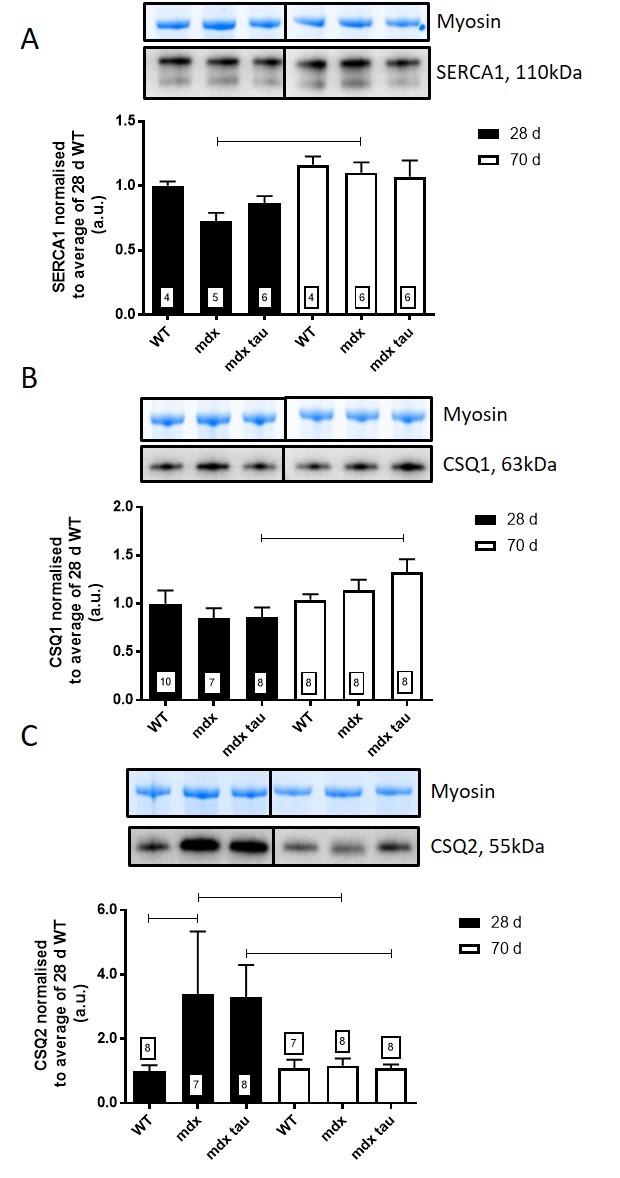
Shown for each panel is the myosin from the Stain Free gel, indicative of total protein (top) and the representative Western blot protein (middle) and quantification of the abundance of SERCA1 (A), CSQ1 (B) and CSQ2 (C) in TA muscle from 28 d (black bars) and 70 d (white bars) WT, mdx and mdx tau mice expressed relative to the 28 d WT. One way ANOVA with Sidak’s post-hoc analyses between relevant groups. Data presented as means + SD with n indicated in respective bars. Lines connecting different bars indicate significance at p<0.05.

**3.7 Effect of taurine supplementation on the abundance of pathologically relevant proteins**

Dystrophin was absent in all mdx groups and present in the WT confirming the phenotype (Fig. 8A). Taurine supplementation had no effect on the abundance of utrophin or myogenin at 28 d (Fig.s 8B, 8C). Utrophin was 180% greater in 28 d mdx mice compared to the 70 d mdx mice (Fig. 8B). Interestingly, myogenin abundance in 70 d mdx tau mice was 450% and 230% greater than 28 d mdx tau and 70 d mdx mice, respectively (Fig. 8C).

**Dystrophin, utrophin and myogenin in 28 and 70 d WT, mdx and mdx tau mice.**
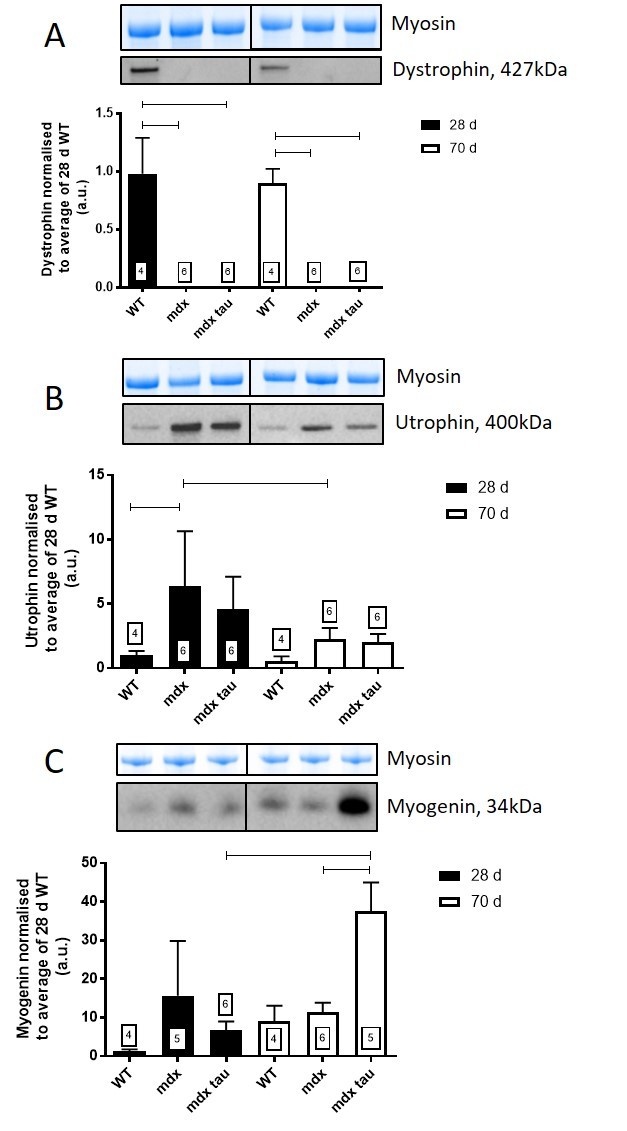
Shown for each panel is the myosin from the Stain Free gel, indicative of total protein (top) and the representative Western blot protein (middle) and quantification of the abundance of dystrophin (A), utrophin (B) and myogenin (C) in TA muscle from 28 d (black bars) and 70 d (white bars) WT, mdx and mdx tau mice expressed relative to the 28 d WT. One way ANOVA with Sidak’s post-hoc analyses between relevant groups. Data presented as means + SD with n indicated in respective bars. Lines connecting different bars indicate significance at p<0.05.

## Discussion

The major aim of this study was to compare the efficacy of taurine supplementation given prior to conception at ameliorating dystrophic symptoms in the 28 and 70 d mdx mouse. Here we have demonstrated taurine to be beneficial during the acute damage phase at 28 d, but not at 70 d where the pathology of the mdx mouse has become stable. These findings support the potential of taurine to act in a protective role in the treatment of DMD, and highlight the importance of age as a key consideration when utilising the mdx mouse when screening for therapeutic supplements and therapies.

Oral taurine supplementation given to mothers, then to weaned pups, successfully elevated taurine content of the TA muscle in 28 d but not 70 d mdx mice. Interestingly, 28 d mdx mice had endogenous levels of taurine similar to WT mice, and 70 d mdx mice had significantly greater muscle taurine content than both WT and mdx tau mice (Fig. 1D). Whilst not strictly comparable due to different methods of analysis, McIntosh et al^28^ previously found that the intramuscular taurine content of 3-6 week old mdx mice was lower than WT mice, although in that study the concentration of taurine was ~10-fold less than the values obtained here (2.17 and 2.61 μmol.g^-1^, in WT and mdx mice, respectively), suggesting that the total pool of taurine was not measured in that study. In line with the current work, intramuscular taurine content was similar in quadriceps muscle from 22 d mdx and WT mice^13^ and 42 d mdx and WT mice (~24 μmol.g^-1^)^17^, although decreased in 28 d mdx compared with WT mice^17^. Whilst we report an age-related increase in TA intramuscular taurine content in mdx mice, in our hands there was no increase in muscle taurine content in 70 d mdx tau mice. When supplementation began at 18 d, 24 days of taurine supplementation in mdx mice increased TA taurine content^16^, suggesting that the lack of taurine increase we observed was due to events in the last four weeks. That taurine did not improve the contractility of mdx muscle at 70 d suggests that the beneficial effects of taurine are not independent of the pathology. Indeed, the lower taurine content in 70 d mdx tau mice suggests there must be a decrease in taurine uptake in those mice. Cozzoli et al^29^ reported elevated muscle taurine content in 8-12 week old taurine supplemented mdx mice compared to mdx controls, although given the 5 week age differential of mice used in that study, it is difficult to interpret further. A further difference from our current study is that those animals had undergone damaging exercise protocols to increase the severity of the mdx phenotype, a commonly used intervention, although it is not known if that intervention may affect the intramuscular taurine content, for example, by the muscle compensating for any taurine loss due to muscle damage, which makes direct comparisons not possible. The similar muscle taurine content in 70 d mdx tau mice which we observed could be due to the relative muscle health of mdx tau mice already seen at 28 d (see discussion on histopathology) possibly carrying through to adulthood (Fig. 1B, Fig. 5A, see mdx tau 28 d).

In this study we found no difference in the abundance of the TauT protein between WT, mdx and mdx tau mice at either 28 d or 70 d. Given the change in intramuscular taurine content, this finding suggests that the activity of the protein is regulated via post-translational modifications. Terrill et al^17^ similarly identified that TauT protein content in quadriceps muscle from non-supplemented mdx mice did not change with age, however TauT protein content was found to be consistently lower than 18, 28 and 42 day age matched WT mice. It should be noted that differences in sample preparation may explain some of the disparity between the two studies and so direct comparison may not be valid. De Luca et al^30^ found an increase in plasma taurine content in 6-8 month old mdx mice suggesting issues with the function of the TauT in uptake or retention in the muscle. Given we have only measured the TauT protein abundance we are not able to comment on any potential difference in the activity of the transporter.

We have uniquely measured in situ force production in 28 d mdx mice with and without taurine supplementation. Investigating contractile characteristics in situ affords many translational benefits. Having nerve and blood flow intact, maintaining normal temperatures and incorporating nerve stimulation provide an ideal situation for assessing isometric force production in a physiologically relevant setting (See SOP in methods). When looking at contractile characteristics in situ, compared with mdx mice there was a significant increase in peak twitch (P_t_) and a mild, yet not significant, increase in maximum force in 28 d mdx tau mice, with no such differences in 70 d mdx and mdx tau mice (Table 1 and Fig. 2A). When expressed as specific force which takes into account the ~23% greater muscle mass, the TA muscles from 28 d mdx mice were weaker than both the WT and mdx tau mice (Table 1, Fig. 2B. In 70 d mice, there was no difference in maximum force between the groups, although the mdx mice had significantly reduced specific force than the WT, but not different from the mdx tau mice (Fig. 2B). A reduction in force production despite an increase in muscle mass would classically be considered as pseudohypertrophy, whereby a large proportion of the muscle is replaced by fat and connective tissue. Rather, visual analysis of muscle from 70 d mdx and mdx tau mice reveals a low occurrence of fibrosis and myofibers of increased size representative of true hypertrophy, and not pseudohypertrophy (Fig 5A – mdx 70 d). Investigating strength relative to muscle size is important as, similar to boys with DMD, regenerating mdx mice can undergo a compensatory hypertrophy of skeletal muscle to circumvent weakness^31^^,^^32^. In absolute terms, muscle from mdx mice has previously been found to be comparative in strength to WT mice, although weaker when expressed as specific force^33^. Different methodologies make it difficult to draw direct comparisons, however previous studies investigating taurine supplementation in mdx mice aged 6-12 weeks have similarly reported it to be efficacious at enhancing grip strength in vivo and in isolated muscle preparation ex vivo^16^^,^^20^^,^^29^.

At 28 d mdx mice produced a greater proportion of their maximum force at lower stimulation frequencies (10 and 20 Hz) compared to the WT, with there being no difference between mdx and mdx tau mice (Fig. 3). There was no phenotype differences apparent in 70 d mice, however across most frequencies measured, the force produced expressed as a percentage of maximum force was typically greater in the 28 d and 70 d mdx mice compared with the age-matched mdx tau mice (Fig. 3). Our finding in the younger mice is consistent with prior studies finding mdx mice to having a more negative mechanical threshold (MT) for excitation-contraction coupling (E-C coupling)^34^. Briefly, E-C coupling takes place when a depolarisation event activates the voltage sensing dihydropyridine receptor (DHPR) which causes Ca^2+^ release from the sarcoplasmic reticulum (SR) via pressing on the ryanodine receptor (RyR). Ca^2+^ subsequently activates the contractile apparatus, stopping when sufficient Ca^2+^ is sequestered back into the SR via the Ca^2+^ ATPase pump (SERCA) where the Ca^2+^ is bound to calsequestrin (CSQ)^35^. There is evidence that mdx mice conduct action potentials normally but subsequent SR Ca^2+^ release is moderately reduced^36^^,^^37^^,^^38^. This reduction may be compensatory, as cytosolic Ca^2+^ content in mdx mice resulting from membrane tears and/or ion channel dysfunction is elevated, thus reducing the amount of Ca^2+^ release required for contraction and resulting in a decrease in the MT^30^. While speculative this allows dystrophic muscle to contract more easily, the deleterious effects associated with disrupted Ca^2+^ homeostasis, such as activation of Ca^2+^ dependent proteases, make ameliorating the MT a desirable outcome when assessing DMD treatment options. In adult mdx mice, taurine has previously been found to ameliorate the MT in vitro, both when applied directly to whole EDL preparations and in EDL muscle fibers from taurine supplemented mdx mice that had undergone damaging exercise to induce the severity of the phenotype^20^^,^^30^. In our in situ model, comparing the percentage of maximum force at a given frequency along with similar abundance of proteins involved in E-C coupling and Ca^2+^ regulation, we found that in both 28 d and 70 d mdx mice, taurine was ineffective at influencing Ca^2+^ homeostasis.

Despite the increase in maximum contractile force in the 28 d mice, taurine had no effect on TTP or ½RT (Table 1) which can be crude indicators of the rates of Ca^2+^ release and re-uptake by the ryanodine receptor (RyR, or calcium release channel) and SERCA pump, respectively. Using western blotting, we report no change in the abundances of RyR (Table 2) and SERCA (Fig. 7) proteins with age or phenotype, although the protein contents were highly variable. The similar abundances of RyR1 and SERCA1 further supports the similar levels of Ca^2+^ homeostasis discussed above. Additionally, our finding in the 70 d mice is consistent with a prior study in slightly younger, 8 week old mdx mice where no differences in these proteins were observed^39^. However, while content levels are consistent, we cannot preclude any impact of taurine on the activities, or efficiencies, of these proteins. Indeed SERCA activity is suppressed in mdx mice as well as double knock-out mice, which are deficient in both dystrophin and utrophin^40^, and it is possible that taurine could improve SERCA activity and warrants further examination in these models. The abundance of CSQ2, a high capacity Ca^2+^ binding protein located in the SR (typically associated with slow twitch muscle), was ~3-fold greater in 28 d mdx compared with WT mice, and was similar to the CSQ2 content in mdx tau mice (Fig. 7). By 70 d of age, the amount of CSQ2 was no longer different between phenotypes (Fig. 7). The increased CSQ2 abundance in 28 d mdx mice may point towards a shift in the muscle isoform, however we found no differences in the abundance of the slow myosin isoform (MHC1, Table 2) or in the abundance of CSQ1, the CSQ isoform dominating in fast-twitch muscle (Fig. 7), suggesting this is not the case. Murphy et al^25^ suggested CSQ2 may have a role in reducing the amount of free Ca^2+^ within the SR, which would prevent SR leak from further exacerbating the high cytosolic Ca^2+^ already associated with dystrophy. Previously the abundance of total CSQ (i.e. CSQ1 and CSQ2) was shown to be reduced in 9 week old mdx mice and it was suggested that this contributes to a reduced Ca^2+^ buffering capacity^41^. We found no such reduction in the abundance of CSQ isoforms in either 28 d or 70 d mice, however it is difficult to compare the findings from the two studies because in the previous work total muscle extract preparation seemingly involved removal of fractions through filtration or centrifugation, which even at low centrifugal force has been shown to result in loss of up to 60% of the total CSQ in cardiac tissue^26^ and (ii) the CSQ isoforms, CSQ1 and CSQ2, were not distinguished from each other as they were here.

In response to the 60 Hz in situ fatigue/recovery protocol 28 d mdx mice were more resistant to fatigue during 180 s of the intermittent low frequency stimulation than WT mice and this was not different in mdx tau mice (Fig. 4). There was no difference observed between the 70 d groups. Generally mdx mice are reported to be highly susceptible to fatigue although this is dependent on mouse age and the nature of the fatigue protocol^31^^,^^33^. Sacco et al 42 repeatedly stimulated the TA of adult female mdx mice at 40 Hz and similar to the current study, reported that mdx mice were more resilient to fatigue than the WT mice. Taken together, our findings suggest that the weaker mdx mice, as measured by a decreased specific force (Fig. 2B), require less ATP and can thus spare energy for further contractions. Due to the smaller muscle size in 28 d mice, it would be expected that a larger percentage of the muscle is recruited during a 60 Hz stimulation compared with the larger 70 d animals, which would be expected to result in the muscle fatiguing more readily, although this was not observed. Interestingly, all 28 d animals, but not 70 d animals, experienced an increase in maximal force production following the fatigue protocol (Fig. 4-right). This potentiation is due to increased phosphorylation of the myosin light chains following repeated stimulation, subsequently making the contractile filaments more sensitive to Ca^2+^ creating more, as well as greater, cross bridging^43^^,^^44^. Given that the supra maximal force post fatigue was also observed in the 28 d WT mice excludes a pathological based explanation for the potentiation, leaving large muscle motor unit recruitment due to small muscle size as a likely explanation. Previously a higher susceptibility to fatigue was reported in mdx mice, but this was assessed in the diaphragm which, unlike hindlimb muscles of these animals that regenerate into adulthood, continues to degenerate throughout the life the mdx^45^. Grip strength of 3-24 week old mdx mice fatigued more rapidly than WT mice^46^ however when considering the avoidance behaviour mdx mice exhibit towards exercise^9^^,^^47^ it is unclear if this data is a true physiological representation of muscular endurance. The fatiguing properties of EDL muscle were reported to be greater at physiological temperature (35^o^C compared with 20^o^C)^48^. Using an in situ protocol we have addressed physiological temperature, kept nerve and blood flow intact and circumvented avoidance behaviour to get a true indication of muscular endurance capacity and at this frequency we found no difference in the fatigability of mdx mice at 70 d of age. Given that fatigue and recovery were not compromised in 70 d mdx mice it was perhaps not surprising to see no difference in mdx tau mice. This is in contrast to studies in rats, where taurine supplementation improved fatigability^21^^,^^49^.

There appears to be a link between increased strength and improved histological profile and visible health in 28 d mdx tau compared with mdx mice, with similar improvements seen with age (Fig. 5). Non-supplemented 28 d mdx mice displayed classic markers of dystrophic damage with large areas of non-contractile and necrotic tissue with a prevalence of recently regenerating, centrally nucleated myofibers. Speculatively, the force deficit seen in the mdx mouse may be due to the loss of healthy, functional contractile tissue seen here. However this may also be due to the high proportion of immature myofibers seen at 28 d, which are a result of the acute onset of myofiber necrosis the model experiences at approximately 21 d^9^. In contrast there is little evidence of active necrosis in 28 d mdx tau mice, which have approximately half the amount of non-contractile tissue seen in the mdx group and subsequently produced approximately twice the amount of force. There was also a decrease in the number of centrally nucleated fibers seen in mdx tau mice when compared to mdx mice. A similar result was found by Terrill and colleagues^13^, when supplementing mdx mice with taurine from 14 days of age, finding a reduction in myofiber necrosis and inflammation, also finding there was little to no marked presence of centrally nucleated myofibers in 22 d juvenile mdx mice. Previously mdx mice with higher taurine content have also been found to have the most effective muscle regeneration^50^. In the current study it is unclear why the large, seemingly mature myofibers in 28 d mdx tau mice are centrally nucleated, despite no evidence of active necrosis or other recent artefacts of muscle damage. It may be explained by some developmental alteration as a result of in utero taurine supplementation or that necrosis occurred at a time point early enough to allow the development of a mature myofiber or that taurine is facilitating a more rapid regenerative response.

In muscle from 70 d mdx and mdx tau mice, there are large, recently regenerated myofibers and it is likely that the central nuclei are an artefact of prior, rather than current damage. Those mice also display a more uniform muscle fiber diameter and a comparable amount of non-contractile tissue to the WT mice, providing an explanation for why, in the most part, they are capable of conferring more mechanical strength than the smaller, recently regenerated myofibers of the 28 d mdx mice (Fig. 5A, compare white space and clumped nuclei in mdx 28 d, and relative fiber size in mdx tau 28 d). Similar to contractile strength, the improved histology seen between mdx and mdx tau mice at 28 d was not evident in mdx and mdx tau mice at 70 d. This was not surprising, as it is well-documented that 10-14 week old mdx mice have little dystrophic phenotype due to the successful regenerative period. The inclusion of this age group in the current study, was to highlight that these mice, unless chronically exercised to aggravate the pathology, as widely used^9^, are not a good dystrophic model, whilst the 28 d mdx mouse presents an appropriate model.

As a measure of myofiber health, we also examined the abundance of myogenin protein, a myogenic regulatory factor involved in skeletal muscle health, development and regeneration. In 28 d mice, the abundance of myogenin was variable and not significantly different between groups of mice; however in 70 d mdx tau mice there was a ~3-fold increase in myogenin protein content compared with mdx mice. Previously it was reported that myogenin was dispensable for skeletal muscle health in adult mdx mice although it appeared to be elevated in response to muscle degeneration in juvenile mdx mice^51^. Certainly, at the mRNA level, compared with WT mice, myogenin was elevated in 21 d mdx mice and seemingly sustained as mice aged to over 12 months, although the latter finding was reported from a total of two animals^52^. Whilst our findings of increased myogenin content in mdx tau mice may indicate improved muscle health in the taurine supplemented 70 d mice, the upregulation of myogenin in 70 d mdx tau occurred despite there being no visible or quantifiable difference in the myofiber health between mdx and mdx tau mice. It is widely recognised that elevated myogenin, along with other markers such as increased acetylcholine receptor abundance are markers of denervation that also occur in ageing muscles^53^. Since denervation of the neuromuscular junctions has been reported in muscle from mdx mice the elevated myogenin in mdx tau animals (at 70 d) may reflect this denervation, rather than being due to a disturbed aspect of myogenesis^54^^,^^55^. Further, the findings might indicate that myogenin levels may not be down regulated in the regenerated muscle from older mdx mice. Given that intramuscular myogenin and taurine contents have independently been associated with healthy muscle development and function, it appears that any synergistic benefit is below the measurable limits of the muscle histopathology and force production.

## Conclusions

The mdx mouse is an invaluable tool to study both the mechanisms of dystrophic damage, and assess potential therapeutic supplements and therapies to remedy the severity of DMD. The age dependent disease severity of the mdx mouse affords the unique opportunity for both the assessment of therapies to prevent or delay dystrophic symptoms (21 - 28 d), as well as treat the established disease state (70 d). This study demonstrates prenatal taurine supplementation to be efficacious at ameliorating muscle weakness and improving histological characteristics in the mdx mouse model of DMD during the acute stage myofibre necrosis seen at 28 d, but not at 70 d where the background pathology is initially mild. The complexity of DMD makes identifying supplements and therapies to target specific pathways difficult. Whilst not able to completely ameliorate the many pathological consequences of DMD, the beneficial effect of taurine seemingly lies in its ability to remedy many of the downstream pathways such as Ca^2+^ handling, anti-inflammation and anti-oxidation simultaneously, cumulatively reducing the severity of the pathology. Considering these findings and that taurine is a cheap, readily accessible and side effect free dietary supplement, we envisage taurine could be administered to pregnant mothers in a protective capacity, reminiscent of folate in the prevention of spinal bifida.

## Corresponding Author

Dr. Robyn M. Murphy

Department of Biochemistry and Genetics, La Trobe Institute for Molecular Science, La Trobe University, Melbourne, VIC 3086, Australia.

Email: r.murphy@latrobe.edu.au

## Competing Interests

There are no competing interests associated with this study.

## Data Availability

The data sets associated with this project are available on figshare: https://doi.org/10.6084/m9.figshare.5331148.
